# Clinical Trials of Active Surveillance of Papillary Microcarcinoma of the Thyroid

**DOI:** 10.1007/s00268-015-3392-y

**Published:** 2016-01-07

**Authors:** Akira Miyauchi

**Affiliations:** Kuma Hospital, Center for Excellence in Thyroid Care, Kobe, Japan; Department of Surgery, Kuma Hospital, 8-2-35 Shimoyamate-dori, Chuo-ku, Kobe, 650-0011 Japan

## Abstract

**Background:**

The incidence of thyroid cancer is increasing globally. This is mainly due to the increase in the detection of small papillary carcinomas, including papillary microcarcinomas (PMC) 1 cm or smaller. It was suggested recently that PMCs are overdiagnosed and overtreated.

**Methods:**

In 1993, the author proposed a clinical trial to compare surgery and observation for low-risk PMC at doctors’ meeting in Kuma Hospital, which was approved and the trial started in the same year. Patients choose immediate surgery or observation. This paper shares our 22-year experience with the active surveillance of more than 2000 patients with low-risk PMC and compares the outcomes of immediate surgery with that of active observation.

**Results:**

The oncological outcomes of these management groups were similarly excellent. In our active surveillance trial on 1235 patients, 8 % of patients showed tumor enlargement by 3 mm or more at 10 years of observation, and 3.8 % of the patients showed novel appearance of lymph node metastasis at 10 years. Patients 40 years or younger tended to show progression of the disease. Patients with these slight progressions of the disease were successfully treated with a rescue surgery. None of the patients in both study groups died of the disease. However, incidences of unfavorable events, such as temporary vocal cord paralysis (VCP) and temporary and permanent hypoparathyroidism, were significantly higher in the immediate surgery group than in the observation group (4.1 vs. 0.6 %, *p* < 0.0001; 16.7 vs. 2.8 %, *p* < 0.0001; and 1.6 vs. 0.08 %, *p* < 0.0001, respectively). Permanent VCP occurred in two of the surgery group.

**Conclusions:**

As a result, although we still offer two options, immediate surgery or observation, to patients with low-risk PMC at Kuma Hospital, we now strongly recommend observation as the best choice.

## Introduction

The incidence of thyroid cancer has been increasing rapidly worldwide, due in major part to improvement of imaging systems. A 2.4-fold increase was reported in the incidence of thyroid cancer in the United States during the 30 years from 1973 to 2002 [1]. This increase was due to the increase in the incidence of papillary carcinoma, and the incidences of follicular, medullary, and anaplastic carcinoma remained stable. Looking more closely at the size of papillary tumors, they found that the incidence of tumors 2 cm or smaller had increased, while that of larger tumor had remained stable. Papillary carcinomas 1 cm or smaller, which are called papillary microcarcinomas (PMC), comprise 49 % of the cases. Although the incidence of thyroid cancer increased 2.4-fold, the mortality from thyroid cancer remained stable. With these results, they concluded that the increase was mostly due to the increase in detection of small papillary cancer, and they suggested the possibility of over-diagnosis and over-treatment. In 2014, they updated their data, showing a 2.9-fold increase in thyroid cancer incidence from 1975 to 2009, while the mortality from thyroid cancer again remained stable [2]. In Korea, the incidence of thyroid cancer increased by 15 times in <20 years from 1993 to 2011 [3]. The increase was again due to the increase in the incidence of papillary carcinoma, and the thyroid cancer mortality remained stable. A similar trend started much earlier in Japan, probably because ultrasound examination and fine needle aspiration biopsy to detect and evaluate thyroid nodules came to be used much earlier in Japan than in the U.S. and Korea.

Regarding PMC, the following facts have been reported. Many autopsy studies on subjects dying of non-thyroidal diseases reported high incidences of latent thyroid cancer [4–6]. If we look at papillary cancer larger than 3 mm, which can be easily detected with ultrasound examination, incidences of 3–5.2 % have been reported [7]. Ultrasound examination allows detection of small thyroid tumors and ultrasound-guided fine needle aspiration (FNA) made diagnosis of these tumors possible, especially for papillary carcinoma. Koji Takebe at Kagawa Cancer Examination Center conducted a screening study using ultrasound examination and ultrasound-guided FNA on subjects who visited his center for the purpose of breast cancer screening. He found that 3.5 % of otherwise healthy Japanese adult women had a small thyroid cancer [8]. This incidence was similar to the incidence of latent thyroid cancer in autopsy studies and more than 1000 times the prevalence, 3.1/100,000, of clinical thyroid cancer in Japanese women at that time. The chances of detection are increasing because of increasing use of imaging studies such as ultrasound, CT scan, MRI, and PET. As a result, the number of patients with PMC is increasing rapidly. This is a worldwide phenomenon. Therefore, the question of how to treat PMC has become a pressing clinical issue.

We do not know the natural history of PMC. One hypothesis is that all PMCs are at an early stage of clinical cancer, which may cause any harm or even kill the host. Another hypothesis is that most PMCs stay small; they are innocent or harmless cancers. Which hypothesis is true? The latter seemed more likely to me, but some PMCs do indeed grow. And of course, all advanced cancers start out as small lesions. The critical question then becomes, “How do we determine which small cancers are likely to cause harm?” Assuming that doing surgery for all PMCs might result in more harm than good, the hypothesis that observation without immediate surgery would provide an answer, and that doing surgery for those who showed slight progression would not be too late was tested in this study.

## Methods and materials

The present author first proposed an observation without immediate surgery clinical trial at a doctors’ meeting of Kuma Hospital in 1993, where it gained approval, and the trial started in the same year [9, 10]. The approach required some strategic thinking regarding diagnosis by FNA, which is very accurate with a positive predictive value of 98 %. In 1993, there were no guidelines for performing FNA. If we did not perform FNA on patients with suspicious small thyroid nodules, these patients might go and see other doctors at other hospitals. These doctors might perform FNA, and might recommend immediate surgery for thyroid treatment. This could be unfortunate for the patients and for the reputation of Kuma Hospital. After discussion on this issue, we decided to perform ultrasound-guided FNA on all suspicious nodules and to share the diagnosis with patients. For patients with high-risk PMC, we recommended surgery. For patients with low-risk PMC, we proposed two options, immediate surgery or observation. We gave the patients the current knowledge regarding PMC such as high incidences of latent thyroid cancer, the results of the thyroid cancer screening study by Dr. Takebe, the prevalence of clinical thyroid cancer and good prognosis of small papillary carcinoma following appropriate surgery, and also the results of our observation trial in later period of the trial. Patients chose one of the options. We followed patients who chose observation with an ultrasound examination 6 months later and once a year thereafter. We recommended surgery if the tumor grew by 3 mm or more or if novel lymph node metastasis appeared.

High-risk PMC is defined as PMC having one or more of the following: lymph node or distant metastasis, extrathyroid extension, high-grade cytology, or growth during a previous observation. We cautiously included tumors located near the recurrent laryngeal nerve or attached to the trachea in the high-risk category. Low-risk PMC was defined as PMC having none of these features. This definition was a kind of theoretical definition. In our actual series of patients with PMC, none of them presented with distant metastasis, and none of them showed high-grade cytology.

**Fig. 1:**
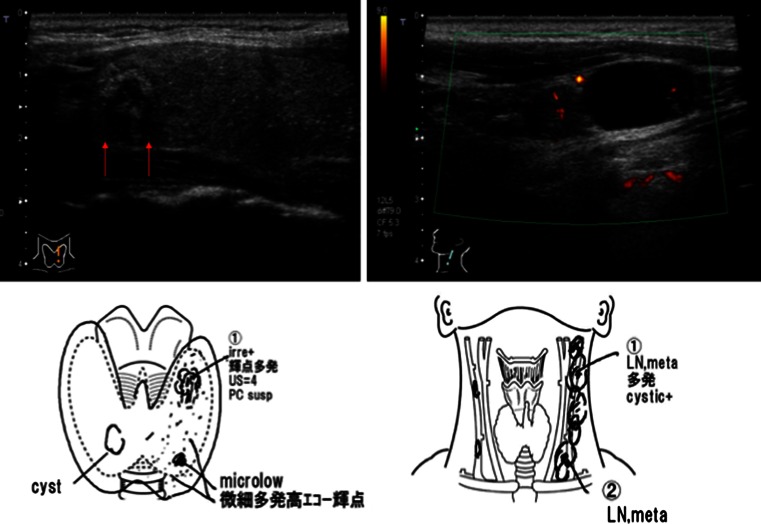
Sonograms and drawings in the report of a 41-year-old woman with papillary microcarcinoma with many nodal metastases in the left lateral neck compartment

**Fig. 2:**
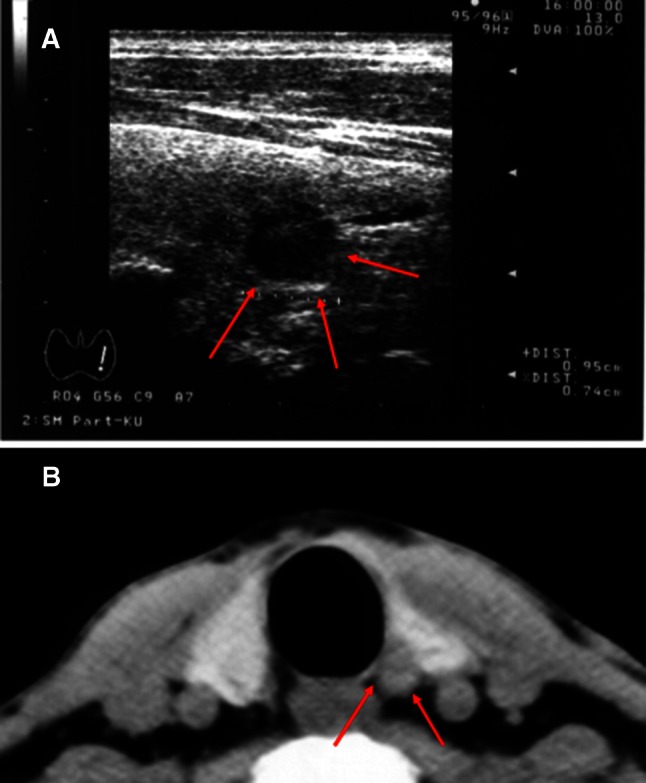
**a** Sonogram of a 74-year-old man with left vocal cord paralysis revealing a small tumor extending from the dorsal surface of his left thyroid lobe. **b** CT scan showing a small tumor located on the course of the left recurrent laryngeal nerve

Patients with PMC may present with cervical lymph node metastases as shown in Fig. 1. These drawings are reports by our sonographers at Kuma Hospital, which are very useful for surgeons in designing surgical operation for the patients. Of course, such patients should be treated appropriately. Total thyroidectomy, central node dissection, and left modified neck dissection were performed for this patient. In another case, a 74-year-old man presented with hoarseness. A laryngoscopy showed left vocal cord paralysis (VCP). An ultrasound examination revealed a small tumor with extrathyroid extension from the dorsal surface of his left thyroid lobe (Fig. 2). A neck CT scan showed a small tumor located exactly on the running course of the left recurrent laryngeal nerve (RLN) (Fig. 2). The FNA revealed papillary thyroid carcinoma. Total thyroidectomy, central node dissection, resection of the left RLN, and reconstruction of the nerve were performed for this patient [11]. The surgical specimen showed PMC with extrathyroid extension, consistent with the imaging studies. These are typical features of high-risk PMC, which should be treated appropriately.

**Fig. 3:**
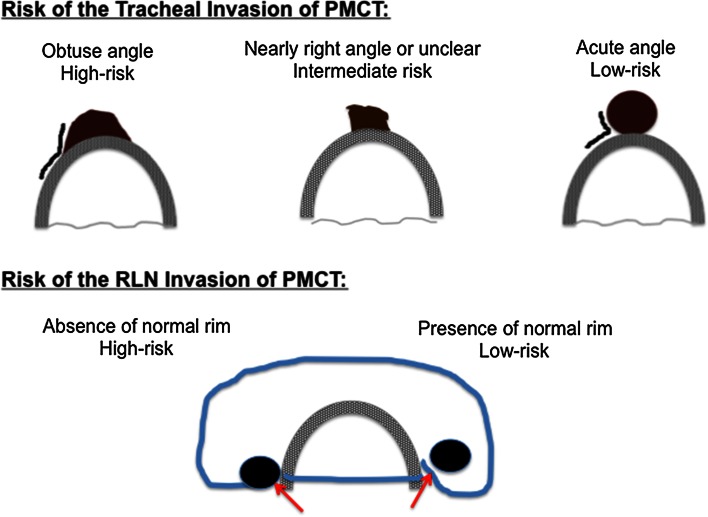
Schematic drawings on the risks of invasion to the tracheal and recurrent laryngeal nerve of papillary microcarcinoma

As previously mentioned, we cautiously included tumors located near the RLN or attached to the trachea in the high-risk category. One of my colleagues, Yasuhiro Ito specifically looked at on this issue and reported at the IAES meeting [12]. Briefly, he found that the risk of tracheal invasion of PMC 7 mm or larger was closely related to the angle formed by the tumor and the trachea. An obtuse angle was associated with high risk of tracheal invasion, a nearly right angle or unclear with intermediate risk, and an acute angle with low risk (Fig. 3). The risk of RLN invasion was also associated with the presence or absence of a normal thyroid rim in the direction closest to the RLN, being high if the normal rim was absent, and low if the normal rim was present (Fig. 3). In our series of PMC-treated surgically, no PMC 6 mm or smaller had significant invasion to the RLN or trachea. We recommended immediate surgery for PMCs with high or intermediate risk features.

## Results and discussion

After 20 years’ experience, we are confident that patients with low-risk PMC features should be considered candidates for active surveillance.

Cancer Institute Hospital in Tokyo started a similar active surveillance study for low-risk PMC 2 years after our trial in Kuma Hospital [13]. Early results of these studies were quite similar; <10 % of PMCs showed an increase in size by 3 mm or more during the observation, about 1 % of the patients showed novel nodal metastasis during the trial, and none of the patients who underwent surgery following the above events had recurrence [9, 13, 14]. With these promising results, Japanese Thyroid Tumor Management Guidelines 2010 published by the Japanese Association of Endocrine Surgeons and the Japanese Society of Thyroid Surgery accepted “Observation for low-risk PMC” as a management [15]. This became the first guideline that accepted observation for low-risk PMC.

In 2014, we reported updated data of our active surveillance trial on 1235 patients with low-risk PMC [16]. In terms of their clinical features, 90 % were female, 40 % were 60 years or older, 14 % were younger than 40 years, 46 % were middle-aged, and 5 % had a family history for papillary carcinoma. Thyroid antibody was positive in 32 % of the patients. Tumor size ranged from less than 5 mm to 10 mm. TSH-suppressive therapies was given in only 4 % of the patients. Twelve percent of the patients had multiple foci, 48 % had associated thyroid nodules, and 6 % were associated with Graves’ disease. The proportion of patients whose PMC showed enlargement by 3 mm or more was 8 % at 10 years of observation. The proportion of the patients whose PMC showed novel appearance of lymph node metastasis was 3.8 % at 10 years. One might think that the appearance of nodal metastasis was a failure of the observation trial. For patients who showed nodal metastasis, we performed total thyroidectomy with central and lateral neck compartment dissection. If these patients had been treated at their presentation, the most likely procedure would have been hemithyroidectomy with or without paratracheal dissection. This procedure would not be able to prevent the appearance of nodal metastasis in the lateral compartment in most cases. Therefore, such patients would require a second surgery, completion thyroidectomy and lateral neck compartment dissection. We think that one surgery is better than two surgeries, since the final outcomes seem similarly good.

Dr. Mazzaferri reported that recurrence rate of thyroid cancer after surgery was high in young and elderly patients and low in middle-aged patients [17]. Although young patients had high recurrence rate, their mortality from thyroid cancer was low, while elderly patients had a high recurrence rate and high mortality rate. We studied the postoperative thyroglobulin status and thyroglobulin-doubling time according to age at surgery in 426 patients with advanced papillary carcinoma who underwent total thyroidectomy in Kuma Hospital [18]. When these patients were treated, only those with advanced disease underwent total thyroidectomy in Kuma Hospital. Detectable serum thyroglobulin postoperatively indicated biochemically persistent disease for these patients. About 40 % each of the patients younger than 40 years and those 60 years or older had biochemically persistent disease postoperatively, while this proportion was 27 % in patients of age 40–59. The proportion of patients with a thyroglobulin-doubling time shorter than 2 years, indicating the presence of rapidly growing hidden metastasis, was low in young (2.4 %) and middle-aged patients (3.9 %), and was high in elderly patients (19.3 %). Interestingly, about half or even more than half of patients with biochemically persistent disease in young and middle-aged patients had a decrease in their serum thyroglobulin values over time (50 and 69 %, respectively), while this proportion was low, at 27 %, in elderly patients. These findings were consistent in some sense with the report by Mazzaferri et al. Age is an important prognostic factor for papillary carcinoma. The UICC/AJCC TNM staging system divides patients with differentiated thyroid carcinoma into young and old groups with the cutoff at 45 years. Our data indicate that dividing patients to three age groups with two cutoffs at 40 and 60 years is more appropriate [18]. At any rate, when I first proposed the observation trial, I knew that the prognosis of elderly patients with clinical thyroid cancer was poorer than younger patients. So, I had some concern about including elderly patients with low-risk PMC in this trial.

However, the actual outcome was different from what expected. PMCs in patients younger than 40 years tended to show an increase in size, while PMCs in patients 60 years or older rarely increased in size. PMCs in the 40–59 year age group were in between (Table 1) [16]. Multivariate analysis for size enlargement of PMC revealed that young age was the only significant factor, and that family history and multiple foci were not significant factors. Patients younger than 40 years tended to show novel lymph node appearance, while middle-aged and elderly patients were unlikely to show nodal metastasis (Table 1) [16]. Multivariate analysis for novel appearance of lymph node metastasis revealed that an age younger than 40 year was the only significant factor, and that family history and multiple foci were not significant factors.

**Table 1:** Proportion of the patients with low-risk papillary microcarcinoma who showed progression of the disease during the active surveillance according to age

	Age at presentation		
	<40 years (%)	40–59 years (%)	≥60 years (%)
Enlargement^a^			
At 5 years	9.1	5.0	4.0
At 10 years	12.1	9.1	4.0
Nodal met^b^			
At 5 years	5.2	1.4	0.5
At 10 years	16.1	2.3	0.5

^a^Enlargement of the tumor by 3 mm or more

^b^Appearance of novel lymph node metastasis

TSH-suppressive therapy was performed for only 51 patients. Of them, only one patient (2 %) showed an increase in the size of PMC, while 57 patients (4.8 %) of the remaining 1187 patients who did not have this therapy showed progression of the disease, tumor enlargement, or nodal metastasis. This difference did not reach significance, possibly due to the small number of patients with TSH-suppressive therapy.

After initiating the observation trial, 186 patients underwent thyroid surgery for various reasons, including a change in the patient’s mind. None of these patients had recurrence of the disease or died from the disease, except for one who developed PMC in the contralateral lobe following hemithyroidectomy. During the active surveillance, 6 patients died of unrelated diseases such as breast cancer, lung cancer, and brain hemorrhage [16].

From the oncological outcome of our active surveillance of low-risk PMC, we can say that those age 40 and older are better candidates for observation than young patients. Although the progression rates are slightly higher in young patients, we think that they also can be candidates for observation, since the final outcome was excellent.

My young colleague Hitomi Oda looked into the negative side of our two management practices for low-risk PMC. She reported on this issue as a poster in the same IAES meeting where this paper was originally presented [19]. In brief, 2153 patients with low-risk PMC were seen from February 2005 to August 2013 in Kuma Hospital. Of them, 974 patients (45 %) chose immediate surgery. Five patients had regional recurrences after the surgery, which were treated successfully. After surgeries, 5 patients died of unrelated diseases. Other than these 5, 969 patients were alive without evidence of disease. 1179 patients (55 %) chose observation. After a certain period of observation, 94 patients underwent thyroid surgery for various reasons. One of them had a regional recurrence, which was treated successfully. All 94 patients were alive and free of the disease. During the active surveillance, 3 patients died of unrelated diseases, and the remaining 1082 patients were alive without cancer progression. Thus, the oncological outcomes of both management groups were both excellent.

However, the incidences of unfavorable events such as temporary VCP, temporary hypoparathyroidism, and permanent hypoparathyroidism were significantly higher in the immediate surgery group than in the active surveillance group (4.1 vs. 0.6 %, *p* < 0.0001; 16.7 vs. 2.8 %, *p* < 0.0001; and 1.6 vs. 0.08 %, *p* < 0.0001, respectively) [19]. Unfortunately, 2 (0.2 %) of the immediate surgery group incurred permanent VCP postoperatively. One was caused by accidental transection of the RLN. Transected nerve ends were anastomosed immediately; however, the vocal cord on that side remained immobile. The other was accidental ligation of the RLN. The ligation thread was released, but VCP persisted. These operations were performed by well-experienced endocrine surgeons at Kuma Hospital, Center for Excellence in Thyroid Care. You might think that surgeries for PMC are simple and easy. However, human errors can occur even while performing simple and easy actions. This is the reality. If surgeries had been done by less experienced surgeons, the incidences of these unfavorable events would have been much higher.

A 49-year-old woman came to see me after she underwent hemithyroidectomy and neck dissection for PMC that was detected by a thyroid cancer screening examination. The surgery was done at another hospital in other prefecture. Immediately after the operation, she got bilateral VCP. Several years later when she caught a cold, pneumonia developed and she required a tracheostomy. None of our patients on the active surveillance developed even unilateral VCP during the observation. The tragedy of bilateral VCP would never have occurred during active surveillance for appropriately selected patients.

**Fig. 4:**
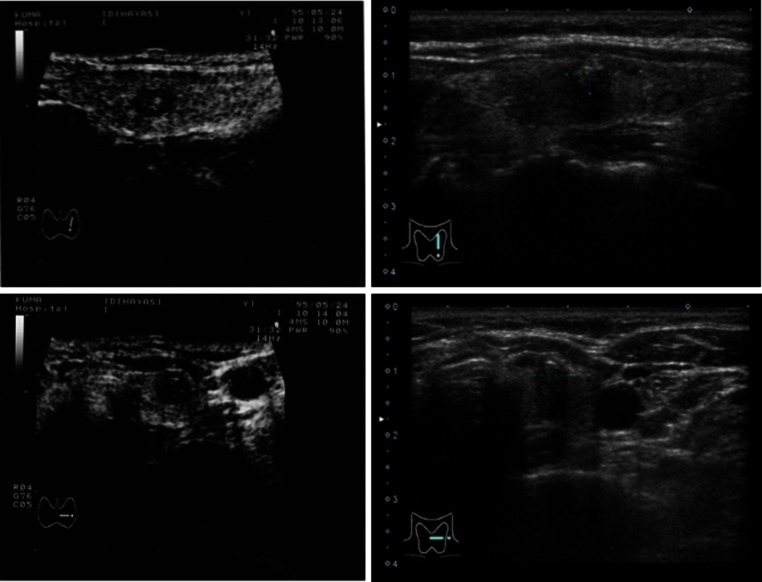
Sonograms of a woman with papillary microcarcinoma at the presentation when she was 46-years old and those 18 years later. Note that the size and shape of the tumor did not change much. Left two images, at presentation; right two, 18 years later

Figure 4 shows sonograms of a woman with PMC at her presentation when she was 46-years old and those taken 18 years later. Although the qualities of the pictures differ each other because of the improvement of ultrasound machine during this long interval, the size and shape of PMC show very little change. This was a very common finding of the active surveillance.

There are limitations to our study. Assignment to observation and immediate surgery groups was not random, but based on the wishes of the patient; hence, some bias might exist in the results.

In conclusion, the oncological outcomes of immediate surgery and active surveillance for PMC were similarly excellent. However, the incidence of unfavorable events was significantly higher in the immediate surgery group than in the active surveillance group. The title being given to my talk was “Clinical Trials of Active Surveillance of PMC of the Thyroid: Is it good?” Now, I say, “Yes! It is good for the patients with low-risk PMC.” Today at Kuma Hospital, although we still offer two options for patients with low-risk PMC, immediate surgery or active surveillance, we recommend active surveillance as the best choice for the patients.
